# Large Orbital Pediatric Intraosseous Hemangioma

**DOI:** 10.1155/2020/5728691

**Published:** 2020-01-02

**Authors:** Colin Bacorn, Lily Koo Lin

**Affiliations:** Department of Ophthalmology and Vision Science, University of California Davis Health, Sacramento, CA, USA

## Abstract

A five-year-old male presenting with progressive right facial swelling underwent multiple biopsies before being diagnosed with a polyostotic frontal-zygomatic primary intraosseous hemangioma. Intraosseous hemangiomas are rare, more frequently afflict adult females, and very rarely involve the orbit. Our case with bony destruction and surrounding soft tissue mass measured 5.3 cm in a child mimicked a more ominous malignancy. This case is unique with its rapid progression and largest reported size, leading to additional challenges such as difficulty in achieving an adequate tissue sample and in the surgical management with respect to significant blood loss in a small child.

## 1. Introduction

Primary orbital intraosseous hemangiomas in children are exceedingly rare [1–5]. Intraosseous hemangiomas are more common in adult females and typically involve the vertebral body and calvarium with the frontal bone making up approximately 45% of reported calvarial cases [6]. Skull tumors may be more common in children while vertebral lesions are identified more frequently in middle aged adults [7]. Involvement of the facial bones is less common [8]. It is important to consider the possibility of this rare tumor preoperatively as life-threatening blood loss during surgical excision can be encountered [9–12].

## 2. Case Presentation

**Figure 1:**
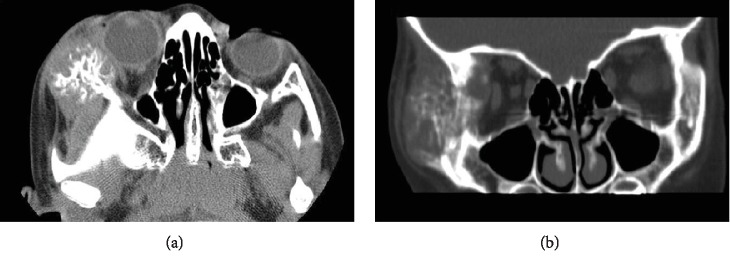
(a) Axial and (b) coronal noncontrast computed tomography (CT) of the orbit of an osteolytic lesion with a large soft tissue component involving the right frontal and lateral orbit and zygomatic arch. A sunburst- or honeycomb-type pattern of new bone formation is seen.

A five-year-old male with no medical or ophthalmologic history presented with one month of a progressively enlarging right facial mass. There was no prior trauma. This painless mass was firm to palpation and measured 3 cm in greatest dimension on initial presentation. Computed tomography (CT) of the orbits revealed a lytic osseous-based mass arising within the right zygoma (Figures 1(a) and 1(b)). The lesion exhibited a nonossified component within the orbit and exerted mass effect on the globe without evidence of scleral invasion. Based on these imaging characteristics, the primary differential diagnosis was Ewing's sarcoma but metastasis was also considered. Further systemic workup with CT of the chest, abdomen, and pelvis as well as a technetium bone scan failed to demonstrate other lesions or evidence of a primary malignancy. Fine-needle biopsy of the mass was inconclusive, showing only compact and woven bone, and the patient was referred for incisional biopsy. Approximately two months following symptom onset, the lesion had continued to enlarge and measured 5 cm in the largest dimension. An incisional biopsy was performed of just the soft tissue component and was again nondiagnostic and without malignant cells.

**Figure 2:**
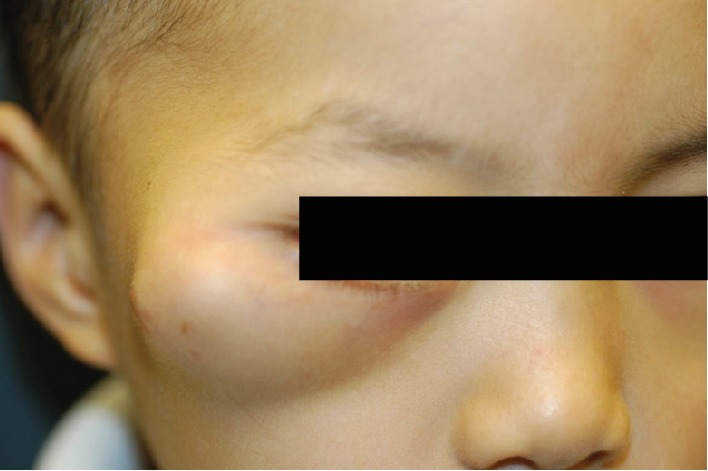
External photograph of patient's right facial mass involving the lateral orbit.

**Figure 3:**
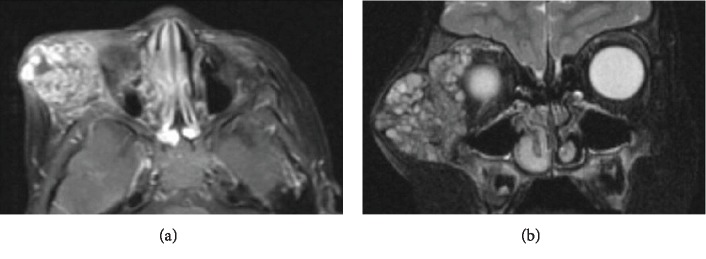
(a) Axial T1-weighted postcontrast fat-saturated magnetic resonance (MR) image shows avid heterogenous enhancement of the soft tissue and intraosseous components. (b) Coronal T2-weighted fat-saturated noncontrast MR image shows the mass with multiple septations/cystic components.

**Figure 4:**
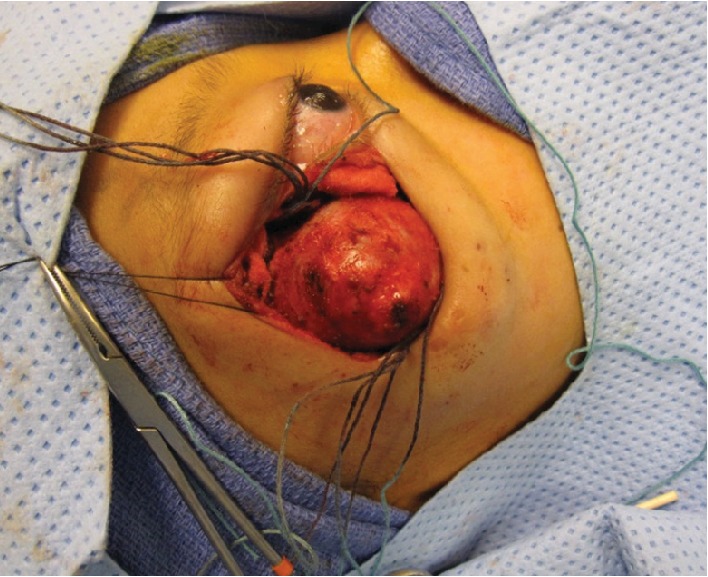
External photograph of the intraoperative gross appearance of the mass.

**Figure 5:**
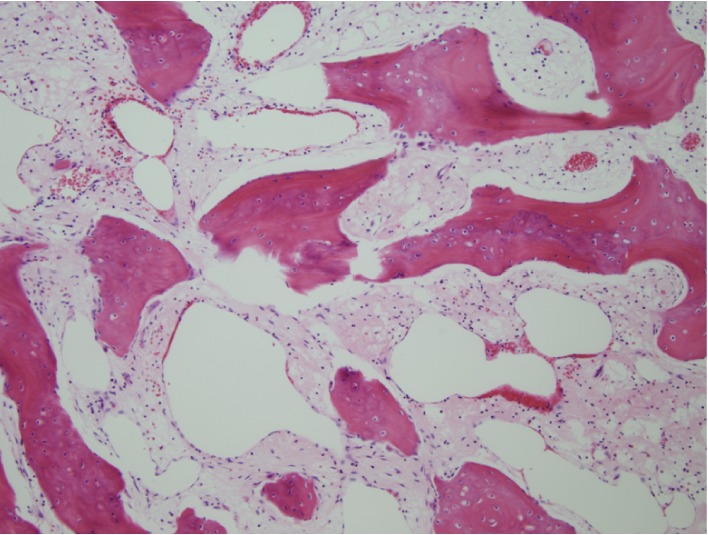
Low-power photomicrograph (original magnification, ×4; hematoxylin & eosin stain) shows dilated thin-walled vessels lined by flattened endothelial cells extending between scattered reactive bone trabeculae.

**Figure 6:**
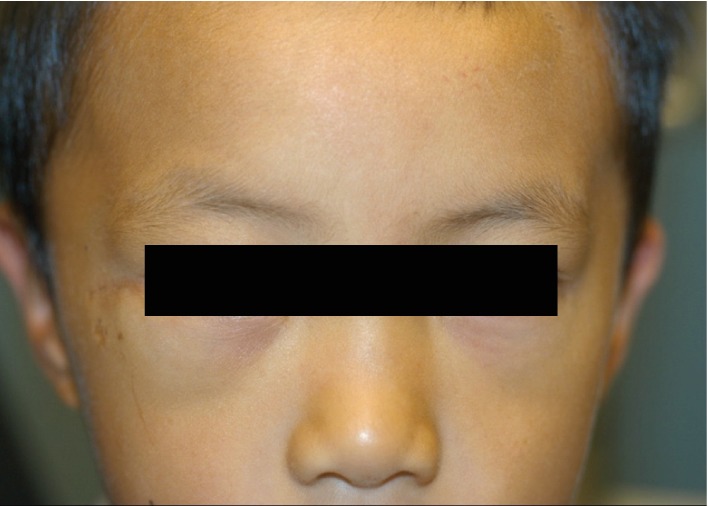
External photograph at the one-month postoperative visit, with resolution of the right orbital mass.

Based on these results, the patient was referred to our oculoplastic service for further management. Ophthalmic examination revealed best corrected visual acuity of 20/25 in the right eye and 20/20 in the left eye with normal stereopsis. The right bony orbital mass was noted (Figure 2) with relative proptosis of 2 mm of the right eye. There was no relative afferent pupillary defect or deficit of extraocular motility. The rest of his ophthalmic examination was normal. Magnetic resonance imaging (MRI) of the orbits was significant for a 5.3 cm mass with suggestion of intralesional vascular channels (Figures 3(a) and 3(b)). A third biopsy was performed via lateral orbitotomy with excision of a 5 cm × 6 cm bony mass. Intraoperatively, the mass was noted to be composed of numerous cystic spaces containing blood vessels and sanguineous material (Figure 4). Complete excision necessitated sacrifice of portions of the lateral and inferior orbital rims. The remaining intact bone was burred smooth, and a reconstructive implant was not necessary. Bone wax, diamond burr, cautery, and TISSEEL fibrin sealant (Baxter Healthcare Corporation, Westlake Village, CA, 91362 USA) were all employed to achieve hemostasis. The patient was typed and cross-matched due to approximately 150 milliliters of blood loss; however, given he remained hemodynamically stable both during and after surgery, ultimately no blood transfusion was administered. The final histopathology revealed reactive intratrabecular spaces containing numerous proliferated, small capillary-sized and dilated thin-walled blood vessels. The single layer of endothelial lining in the proliferating capillary vessels and blood-filled channels within bone confirmed a diagnosis of interosseous capillary hemangioma (Figure 5). Cytogenetic testing revealed normal karyotype supporting a diagnosis of intraosseous hemangioma. Postoperative examination demonstrated acceptable cosmesis (Figure 6). The patient has maintained best corrected visual acuity of 20/20 OD and 20/20 OS with full extraocular motility and without clinical evidence of recurrence eight years following excision.

## 3. Discussion

In children, a number of malignant orbital lesions can present with bone destruction such as Ewing's sarcoma, metastasis (osteosarcoma, neuroblastoma), Langerhans cell histiocytosis, leukemia, and rhabdomyosarcoma [1–5]. Although benign tumors such as fibrous dysplasia, juvenile ossifying fibromas, and intraosseous hemangiomas can demonstrate osteolytic activity, often, the initial goal is ruling out malignancy as was true in our case.

Primary orbital intraosseous hemangiomas in children are extremely rare. A recent case report and literature review identified 49 reported cases of zygomatic intraosseous hemangioma; only five of the patients were under the age of 18 at diagnosis and only seven were noted to have ocular findings [11]. None of these cases documented a lesion of our size (5 cm × 6 cm) in the zygomatic location. A separate study of 24 pediatric cases of cranial intraosseous hemangioma identified only four lesions with invasion into the orbit [13].

Clinically, these tumors often present as a subacute to chronic enlarging, firm, mass which may or may not be painful [14]. Occasionally, patients demonstrate multiple simultaneous lesions, although this is rare in the bones of the skull (10-15%) [15]. More rapid enlargement following trauma has also been reported. The diagnosis is often suggested by radiographic features including the classic description of a lytic lesion with “soap-bubble” or “sunburst” or “honeycomb” appearance; however, this pattern can be seen with other osteolytic etiologies as well [12]. MRI characteristics vary based on the tumor's fat composition and venous flow, but generally, these lesions exhibit either a high (high fat) or intermediate to low (low fat) T1-weighted signal and a heterogeneous, hyperintense T2-weighted signal and contrast enhancement [15–17].

Fine-needle aspiration is technically difficult in these bony tumors and may lack sufficient diagnostic accuracy as our case required excisional biopsy for diagnosis. Needle biopsy can disrupt the thin-walled blood vessels, so the specimen is nonspecific. Biopsy specimens without incorporating the bony component can also be nondiagnostic. Histologic subtypes have been classified as either cavernous, capillary, mixed, or scirrhous on the basis of the size of the vascular spaces and amount of connective tissue within the lesion [11, 18]. While the diagnosis is often suspected on imaging features and gross examination intraoperatively or of the resected specimen, histopathologic examination is useful in differentiating these tumors from other vascular malformations and immunohistochemical analysis for Factor VIII, vimentin, CD31, and GLUT-1 may be appropriate in select cases [12, 19, 20].

Treatment is typically total surgical excision with or without preoperative embolization [16, 21–25]. Given the age of our patient and both the invasiveness of angiography and the need for general anesthesia for the study, preoperative angiography was not pursued. Significant blood loss in the surgical resection of intraosseous hemangiomas has been well documented [9–12]. Our small patient's body weight was 19.3 kilograms at the time of his surgery, with an estimated blood volume (EBV) of 1351 milliliters (EBV = body weight (19.3 kg) × average blood volume of child (70 ml/kg)). Children may require red blood cell transfusion during or after surgery for an acute blood loss of greater than 10-15% EBV [26]. Our patient lost about 150 milliliters of blood intraoperatively, which placed him within this range. Given that he remained hemodynamically stable, and that transfusing red blood cells especially in children is not without risk, he did not ultimately receive a transfusion. However, this should be a consideration when surgically resecting intraosseous hemangiomas of this size in a small child. When lesions are inaccessible, or otherwise unresectable, radiotherapy has also been employed [27–29]. As in our case with 8-year follow-up, recurrence following complete excision is uncommon [29, 30]. Large pediatric intraosseous hemangiomas of the orbit are rare but should be remembered in the differential diagnosis of osteolytic lesions as surgical removal requires preoperative considerations such as the possibility of significant blood loss.

## References

[1] Shields J. A., Shields C. L., Scartozzi R. (2004). Survey of 1264 patients with orbital tumors and simulating lesions: the 2002 Montgomery Lecture, part 1. *Ophthalmology*.

[2] Bonavolontà G., Strianese D., Grassi P. (2013). An analysis of 2,480 space-occupying lesions of the orbit from 1976 to 2011. *Ophthalmic Plastic and Reconstructive Surgery*.

[3] Castillo B. V., Kaufman L. (2003). Pediatric tumors of the eye and orbit. *Pediatric Clinics of North America*.

[4] Alkatan H. M., Al Marek F., Elkhamary S. (2019). Demographics of pediatric orbital lesions: a tertiary eye center experience in Saudi Arabia. *Journal of Epidemiology and Global Health*.

[5] Rao A. A., Naheedy J. H., Chen J. Y.-Y., Robbins S. L., Ramkumar H. L. (2013). A clinical update and radiologic review of pediatric orbital and ocular tumors. *Journal of Oncology*.

[6] Yang Y., Guan J., Ma W. (2016). Primary intraosseous cavernous hemangioma in the skull. *Medicine*.

[7] Philpott C., Wray A., Mac Gregor D., Coleman L. (2012). Dural infantile hemangioma masquerading as a skull vault lesion. *American Journal of Neuroradiology*.

[8] Myadam S., Kishan V., Deepa A., Shri Puja K., Divya R. K. (2016). Intraosseous hemangioma of the zygomatic bone: a rare site for hemangioma. *Medical Journal, Armed Forces India*.

[9] Yu L., Cai L., Yu G. R., Zeng Z. H., Tao S. X. (2009). Solitary giant hemangioma of the humerus. *Orthopedics*.

[10] Charles N. C., Lisman R. D. (2002). Intraosseous hemangioma of the orbit. *Ophthalmic Surgery and Lasers*.

[11] Powers D. B., Fisher E., Erdmann D. (2017). Zygomatic intraosseous hemangioma: case report and literature review. *Craniomaxillofacial Trauma and Reconstruction*.

[12] Madge S., Simon S., Abidin Z. (2009). Primary orbital intraosseous hemangioma. *Ophthalmic Plastic and Reconstructive Surgery*.

[13] Prasad G. L., Pai K. (2018). Pediatric cranial intraosseous hemangiomas: a review. *Neurosurgical Review*.

[14] Marcinow A. M., Provenzano M. J., Gurgel R. K., Chang K. E. (2012). Primary intraosseous cavernous hemangioma of the zygoma: a case report and literature review. *Ear, Nose, & Throat Journal*.

[15] Kiratli H., Orhan M. (1998). Multiple orbital intraosseous hemangiomas. *Ophthalmic Plastic and Reconstructive Surgery*.

[16] Suzuki Y., Ikeda H., Matsumoto K. (2001). Neuroradiological features of intraosseous cavernous hemangioma-case report. *Neurologia Medico-Chirurgica*.

[17] Rigopoulou A., Saifuddin A. (2012). Intraosseous hemangioma of the appendicular skeleton: imaging features of 15 cases, and a review of the literature. *Skeletal Radiology*.

[18] Eliot C. A., Castle J. T. (2010). Intraosseous hemangioma of the anterior mandible. *Head and Neck Pathology*.

[19] Kadlub N., Dainese L., Coulomb-L’Hermine A. (2015). Intraosseous haemangioma: semantic and medical confusion. *International Journal of Oral and Maxillofacial Surgery*.

[20] Yang G., Li C., Chen X. (2014). Large capillary hemangioma of the temporal bone with a dural tail sign: a case report. *Oncology Letters*.

[21] Choi J. S., Bae Y. C., Kang G. B., Choi K.-U. (2018). Intraosseous hemangioma of the orbit. *Archives of Craniofacial Surgery*.

[22] Brandner J. S., Rawal Y. B., Kim L. J., Dillon J. K. (2018). Intraosseous hemangioma of the frontal bone. Report of a case and review of the literature. *Journal of Oral and Maxillofacial Surgery*.

[23] Wu C.-Y., Huang H.-M., Chen D.-C., Cho D.-Y., Wei S.-T. (2016). Primary intraosseous hemangioma of the orbital roof: a pitfall of surgery. *The Journal of Craniofacial Surgery*.

[24] Koybasi S., Saydam L., Kutluay L. (2003). Intraosseous hemangioma of the zygoma. *American Journal of Otolaryngology*.

[25] Park B. H., Hwang E., Kim C. H. (2013). Primary intraosseous hemangioma in the frontal bone. *Archives of Plastic Surgery*.

[26] Rachel H. (2009). Transfusion principles in children. *Anaesthesia & Intensive Care Medicine*.

[27] Singh U., Kalavakonda C., Venkitachalam S., Patil S., Chinnusamy R. (2019). Intraosseous hemangioma of sella: case report and review of literature. *World Neurosurgery: X*.

[28] Rios Dias G. D., Velasco Cruz A. A. (2004). Intraosseous hemangioma of the lateral orbital wall. *Ophthalmic Plastic and Reconstructive Surgery*.

[29] Perugini M., Renzi G., Gasparini G., Cerulli G., Becelli R. (2004). Intraosseous hemangioma of the maxillofacial district: clinical analysis and surgical treatment in 10 consecutive patients. *The Journal of Craniofacial Surgery*.

[30] Gupta T., Rose G. E., Manisali M., Minhas P., Uddin J. M., Verity D. H. (2013). Cranio-orbital primary intraosseous haemangioma. *Eye*.

